# Trends and hotspots of stereoelectroencephalogram from 2002 to 2023: a bibliometric analysis

**DOI:** 10.3389/fneur.2024.1464657

**Published:** 2024-12-17

**Authors:** Tianren Wang, Hengxin Dong, Kaiwei Li, Tao Feng, Yanfeng Yang, Sichang Chen, Di Lu, Penghu Wei, Yongzhi Shan, Guoguang Zhao

**Affiliations:** ^1^Department of Neurosurgery, Xuanwu Hospital, Capital Medical University, Beijing, China; ^2^China International Neuroscience Institute (CHINA-INI), Beijing, China; ^3^Institute for Brain Disorder, Beijing, China

**Keywords:** SEEG, bibliometrics, epilepsy, electrophysiology, neurosurgery

## Abstract

**Background:**

Stereoelectroencephalography (SEEG), as a minimally invasive method that can stably collect intracranial electroencephalographic information over long periods, has increasingly been applied in the diagnosis and treatment of intractable epilepsy in recent years. Over the past 20 years, with the advancement of materials science and computer science, the application scenarios of SEEG have greatly expanded. Bibliometrics, as a method of scientifically analyzing published literature, can summarize the evolutionary process in the SEEG field and offer insights into its future development prospects.

**Methods:**

This article selected all the literature records retrieved on November 4, 2024, from the Web of Science Core Collection (WoSCC). The search terms were as follows: “Stereo-electroencephalography” or “Stereo electroencephalography” or “Stereo-EEG” or “Stereo EEG” or “SEEG.” The document types included were research articles and reviews. For analysis, VOSviewer, CiteSpace, and the R package “bibliometrix” were employed to analyze various aspects of the SEEG field, including authors, institutions, countries and regions, and research hotspots.

**Results:**

We reviewed a total of 1,383 non-duplicate literature records from 2002 to 2023, including 1,241 research articles, 116 review articles and 26 letters. Observing the annual publication trends, there has been an overall increase since 2002. The most influential journal in this field is *Epilepsia*. Other journals with considerable impact include *Clinical Neurophysiology*, *Epileptic Disorders*, *Epilepsy Research*, *NeuroImage*, and *Epilepsy & Behavior*. The top 5 most influential scholars are Bartolomei F, Tassi L, Nobili L, Russo GL, and Mc Gonigal A. As for the analysis of countries and regions, France occupies a leading position in this field with its early start, while China and the United States have also emerged as focal points since 2020. Research on SEEG has expanded beyond its initial use for localizing epileptic foci and thermo-coagulation treatments and have been employed as a medium to facilitate real-time prediction of epileptic seizures and enabling the exploration of brain network connectivity.

**Conclusion:**

As a minimally invasive tool for collecting intracranial electroencephalographic signals, SEEG continues to offer vast potential for development and application. Advances in electrode materials and robotic-assisted stereotactic techniques, have enabled SEEG to simultaneously sample multiple brain regions, acquire electrical signals from deep brain structures. These advantages significantly enhance the precision of epileptic focus localization in diagnosis and treatment, addressing the limitations of subdural electrodes. Through bibliometric analysis, this paper traces the developmental trajectory of SEEG and identifying key technological milestones, thereby providing a reference for scholarly research directions.

## Introduction

Stereoelectroencephalography (SEEG) is a minimally invasive diagnostic approach used in preoperative evaluation for patients with drug-resistant epilepsy. This technique involves inserting intracranial electrodes via stereotactic methods to record and analyze intracranial EEG activity or induce seizures through exogenous electrical stimulation to identify the epileptogenic zones in patients with focal epilepsy. SEEG was first established in the 1950s at Saint Anne Hospital in France (1). Subsequently, in the 1960s, French researchers Talairach and Bancaud applied and promoted a three-dimensional stereotactic protocol for implanting deep brain electrodes, significantly expanding the application of SEEG in epilepsy treatment processes (2). An “anatomical-electroclinical correlation” comprehensive evaluation framework was also proposed by them based on intracranial electrical signals, which has become a crucial paradigm in the current treatment process for drug-resistant epilepsy.

SEEG offers unique advantages in recording intracranial EEG. Compared to subdural electrodes placed via craniotomy, SEEG utilizes intracerebral electrodes implanted via twist drill—allowing for broader spatial coverage including deep brain regions (mesial temporal lobe, insula, thalamus, sulcal depths, midline structures) for longer duration with fewer complications to resolve the localization of the epileptogenic zone (3–5). Furthermore, the electrode configuration of SEEG also enables the application of specific power and frequency electric currents. On the one hand, low-power, short-duration electrical pulses directly applied to the cerebral cortex can be used to induce epileptic seizures (6–8). This can also be utilized to map functional areas to determine the extent of surgical resection (9, 10). On the other hand, high-power, long-duration currents can be used in radiofrequency thermocoagulation to ablate suspected epileptogenic regions identified during SEEG monitoring, allowing for seizure reduction assessment before a more definitive resection if seizures recur (11–13).

In recent years, advancements in materials science and the maturity of stereotactic techniques have further expanded the boundaries of SEEG in epilepsy treatment. These developments provide crucial means for identifying intracranial EEG characteristics and enriching brain network theories.

Bibliometric analysis is a mature and rigorous mathematical method used to analyze a large volume of scientific literature. It can guide future clinical and research efforts in SEEG by providing a quantitative understanding of scientific trends and citation patterns. Additionally, this method can promote collaboration and guidance among scholars by identifying influential authors and institutions. Furthermore, bibliometric analysis can also be used to track the dissemination of new therapeutic theories and assess the effectiveness of treatments aimed at improving prognosis. To date, there has been no specific bibliometric analysis of SEEG technology. Through bibliometric analysis, this article will delve into the evolutionary path and development history of SEEG technology in the diagnosis and treatment of epilepsy, as well as surgical interventions for drug-resistant epilepsy.

This study outlines the following research questions for a comprehensive review of SEEG, including: (1) What is the historical development path of SEEG technology? (2) What advancements have been made in SEEG technology to date? (3) What are the promising directions for future research in SEEG? (4) Who has played a key role in the research of SEEG? Who are the potential mentoring experts and collaborators? (5) What are the important journals, institutions, and countries in the field of SEEG research?

## Materials and methods

We conducted a comprehensive and thorough search of the Web of Science Core Collection (WoSCC). The edition was Science Citation Index Expanded (SCI-EXPANDED). According to the Medical Subject Headings (MeSH) terminology of the National Library of Medicine, the following search terms were used: TS = (“Stereo-electroencephalography”) OR TS = (“Stereo electroencephalography”) OR TS = (“Stereo-EEG”) OR TS = (“Stereo EEG”) OR TS = (“SEEG”). Since Russo and colleagues published a review with SEEG in the clinical evaluation of epilepsy in 2008, detailing its principles and morbidities in epilepsy diagnosis and treatment (14), and considering that the annual publication volume in this field before 2002 was relatively low, this may have a considerable impact on evaluating the trends in publication volume. Therefore, we compiled all articles published from 2002 to 2023, limiting the document types to articles, case reports, and reviews, without imposing any language restrictions. Due to the maximum record limit of 500 per export file in the database, we exported the results along with complete citation records into three separate text files, each named “download_***.txt,” where “***” represents the number of the literature orders contained within that file. These files include basic information about the literature, such as author names (AU and AF), titles (TI), publication names (SO), languages (LA), document types (DT), abstracts (AB), keywords (DE), and cited references (CR). Articles that did not meet the research objectives or were duplicates were independently removed by two researchers.

We employed multiple platforms for bibliometric analysis, including VOSviewer, CiteSpace, and the R package “bibliometrix.” VOSviewer (version 1.6.18) is a fast and freely available software program developed for constructing and viewing bibliometric maps (15). This software is used for co-authorship, co-occurrence, and co-citation analyses. Additionally, we conducted a thorough analysis of the retrieved documents from three perspectives: authors, organizations, and countries. This platform provided users with network visualization, overlay visualization, and density visualization of the data to be analyzed. CiteSpace (version 6.1 R3 basic) features robust mathematical capabilities for calculating centrality, link strength, cluster exploration, and citation frequency (16). Various parameters in the control panel allow users to customize the visualization results, including color maps, burst detection, clusters, labels, layouts, and views. The “bibliometrix” package, first released in 2016, was applied in this study through its web interface “biblioshiny” on R Studio version 1.2.5042. This platform was used to offer specialized perspectives through visualizations such as word clouds, thematic maps, and related topic dendrograms (17). The package also provides rigorous quantitative evaluations with multiple parameters, including h-index and g-index. The h-index, also known as the Hirsch index, is a metric that indicates that an author or a journal has h papers each cited at least h times. The g-index, on the other hand, is a measure for an author or a journal that indicates the top g articles, sorted by citation count, have received citations no less than g squared, but the (g + 1)th article has citations not exceeding (g + 1) squared (18). Both indices are used to represent the level of influence of authors or journals. Higher values for these metrics suggest a greater impact and influence of the evaluated entity.

## Results

### Analysis of publications

**Figure 1:**
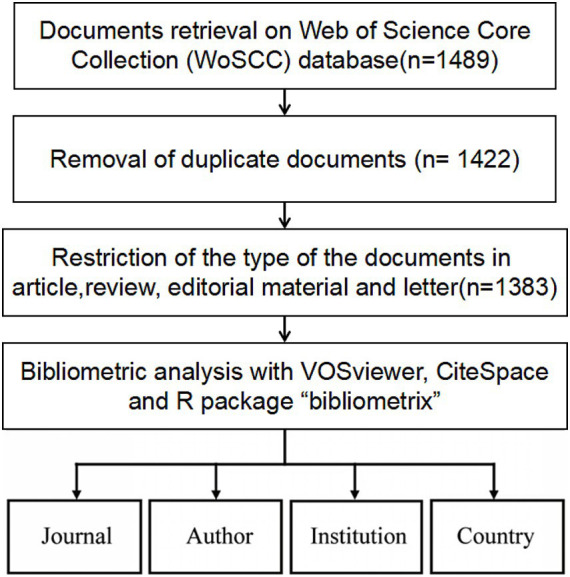
The process for selecting literature for inclusion in the analysis.

**Figure 2:**
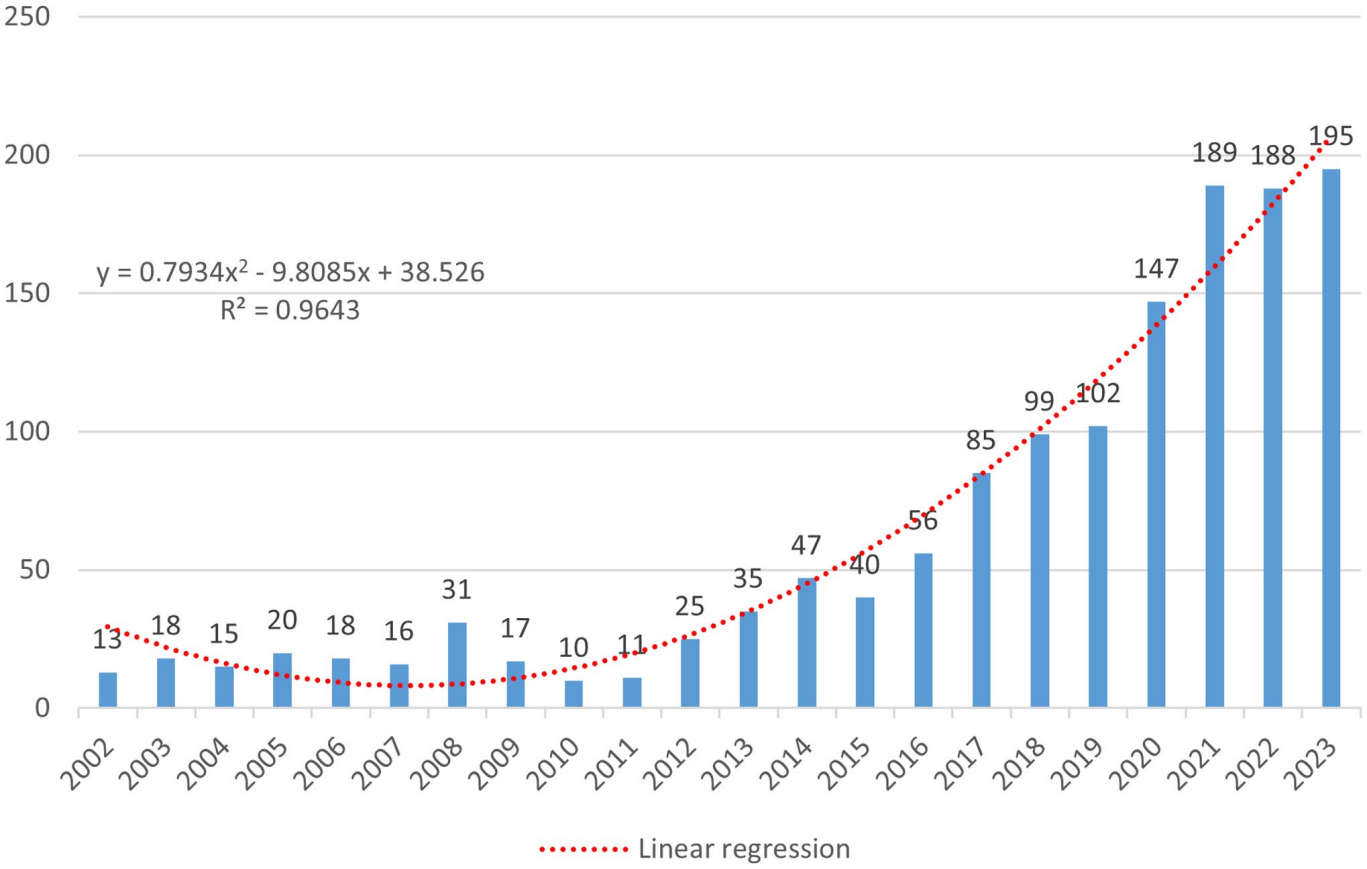
The figure shows the number of relevant publications on the subject published annually over time, with the horizontal axis representing the year and the vertical axis representing the number of publications. The formula is a linear regression model constructed based on the number of annual publications, where *x* denotes the sequential order of the years. For instance, if 2002 is the starting year, then *x_2002_* = 1. The regression model is represented by a red dashed line in the image. *R*^2^ is the coefficient of determination for the linear regression model, and in this model, *R*^2^ = 0.9643.

A total of 1,489 records were retrieved, including 1,299 articles, 161 systematic reviews, and 29 letters. After deduplication by Citespace and manual removal of duplicates, 1,383 unique records were included in the study, comprising 1,241 research articles, 116 review articles, and 26 letters (Figure 1). The annual publication volume in the SEEG field has generally shown an upward trend and peaked in 2023. During the entire trend curve, the steepest slope occurred between 2016 and 2017, where the annual publication volume increased from 56 to 85 papers, reached a growth rate of 51.8%. Since then, the number of studies on SEEG has continued to climb each year, reaching the peak of publication volume in 2023 (Figure 2). It can be said that the popularity of SEEG reached its peak in these 2 years and shows a trend of continued development. SEEG is increasingly appearing in various types of brain science research, and the academic interest in using SEEG for neuroscience research remains high.

To predict the number of publications, a linear regression model was constructed based on the annual number of publications; the formula is as follows (Figure 2):

$y=0.7934x^{2}-9.8085x+38.526$ where x represents the sequential order of the year. The function curve derived from this formula is represented by a red dashed line in the image. According to this fitting formula, we can predict that the publication volume of articles with SEEG as a keyword will continue to grow in the coming years and is likely to exceed 200 articles in 2024. Additionally, the following table lists the top 10 most cited articles (Table 1). It is important to note that in order to more comprehensively assess an article’s overall impact, the calculation of citation counts is not limited solely to articles in the SEEG field. Furthermore, to eliminate the impact of the year of publication on citation counts, this study also calculated the annual average number of citations per article. The article by TASSI L as the first author (19), which uses SEEG to delineate the electrophysiological characteristics of focal cortical dysplasia and accomplishes its classification, received the highest number of citations. Additionally, according to the betweenness centrality calculated by Citespace, an article by Cardinale F as the first author (20), which evaluates the safety, efficacy, and accuracy of SEEG, has been identified as the most important publications in the field. This article, which follows up on five hundred SEEG procedures, confirms the crucial role of SEEG in the preoperative assessment process for intractable epilepsy and provides clinical evidence supporting its widespread application. This highlights SEEG’s significant contribution to enhancing the precision and outcomes of epilepsy treatment strategies.

**Table 1:** The top 10 most cited publications on SEEG.

Title	Authors	Sources	Total citations	Citation per year
Focal cortical dysplasia: neuropathological subtypes, EEG, neuroimaging and surgical outcome	Tassi L et al.	*Brain*	493	24.65
Stereoelectroencephalography: surgical methodology, safety, and stereotactic application accuracy in 500 procedures	Cardinale F et al.	*Neurosurgery*	362	40.22
Clinical manifestations of insular lobe seizures: a stereo-electroencephalographic study	Isnard J et al.	*Epilepsia*	356	19.78
The role of corticothalamic coupling in human temporal lobe epilepsy	Guye M et al.	*Brain*	251	15.69
Stereoelectroencephalography in the presurgical evaluation of focal epilepsy: a retrospective analysis of 215 procedures	Cossu M et al.	*Neurosurgery*	239	14.06
Epileptic fast intracerebral EEG activity: evidence for spatial decorrelation at seizure onset	Wendling F et al.	*Brain*	193	10.16
Decoding of four movement directions using hybrid NIRS-EEG brain-computer interface	Khan MJ et al.	*Frontiers in Human Neuroscience*	187	23.38
Technique, Results, and Complications Related to Robot-Assisted Stereoelectroencephalography	Gonzalez-Martinez J et al.	*Neurosurgery*	183	30.5
Stereoelectroencephalography in presurgical assessment of MRI-negative epilepsy	McGonigal A et al.	*Brain*	178	11.87
The Virtual Epileptic Patient: Individualized whole-brain models of epilepsy spread	Jirsa VK et al.	*Neuroimage*	175	35

### Analysis of journals

The applications of SEEG and the disciplines it involves can be discerned from the types of journals that have published related literature. The analyzing results can also provide guidance for scholars looking to select appropriate journals for their research publications. Using the R package biblioshiny, we obtained statistical results on the publication volumes of various journals. The table shows the top 10 journals contributing most to the dissemination of SEEG research findings. The journal with the highest number of publications in the past 20 years is *Epilepsia* (*n* = 94), and it also leads in terms of the h-index (*n* = 36) and g-index (*n* = 60). Other journals with more than 40 publications include *Clinical Neurophysiology*, *Epileptic Disorders*, *Epilepsy Research*, *NeuroImage*, and *Epilepsy & Behavior* (Table 2).

**Table 2:** The top 10 journals by H-index on SEEG.

Rank	Sources	H-index	G-index	NP	JCR	IF
1	*Epilepsia*	36	60	94	Q1	5.6
2	*Brain*	25	28	28	Q1	14.5
3	*Clinical Neurophysiology*	21	35	68	Q3	4.7
4	*Neuroimage*	21	38	45	Q2	5.7
5	*Epilepsy Research*	18	27	48	Q4	2.2
6	*Epileptic Disorders*	16	29	55	Q4	2.3
7	*Journal of Neurosurgery*	15	29	35	Q2	4.1
8	*Epilepsy & Behavior*	14	20	41	Q3	2.6
9	*Neurosurgery*	12	15	15	Q2	4.8
10	*Neurochirurgie*	11	16	18	Q4	1.6

### Analysis of authors

Author analysis can help us understand who the key contributors are in the field of SEEG research, as well as the collaboration patterns among scholars in previously published articles. By conducting a statistical analysis of all articles published by each author, the top 10 authors in terms of the total number of published papers are Bartolomei F, Tassi L, Nobili L, Russo GL, McGonigal A, Francione S, Chauvel P, Cardinale F, Kahane P, and Mai JH.

**Figure 3:**
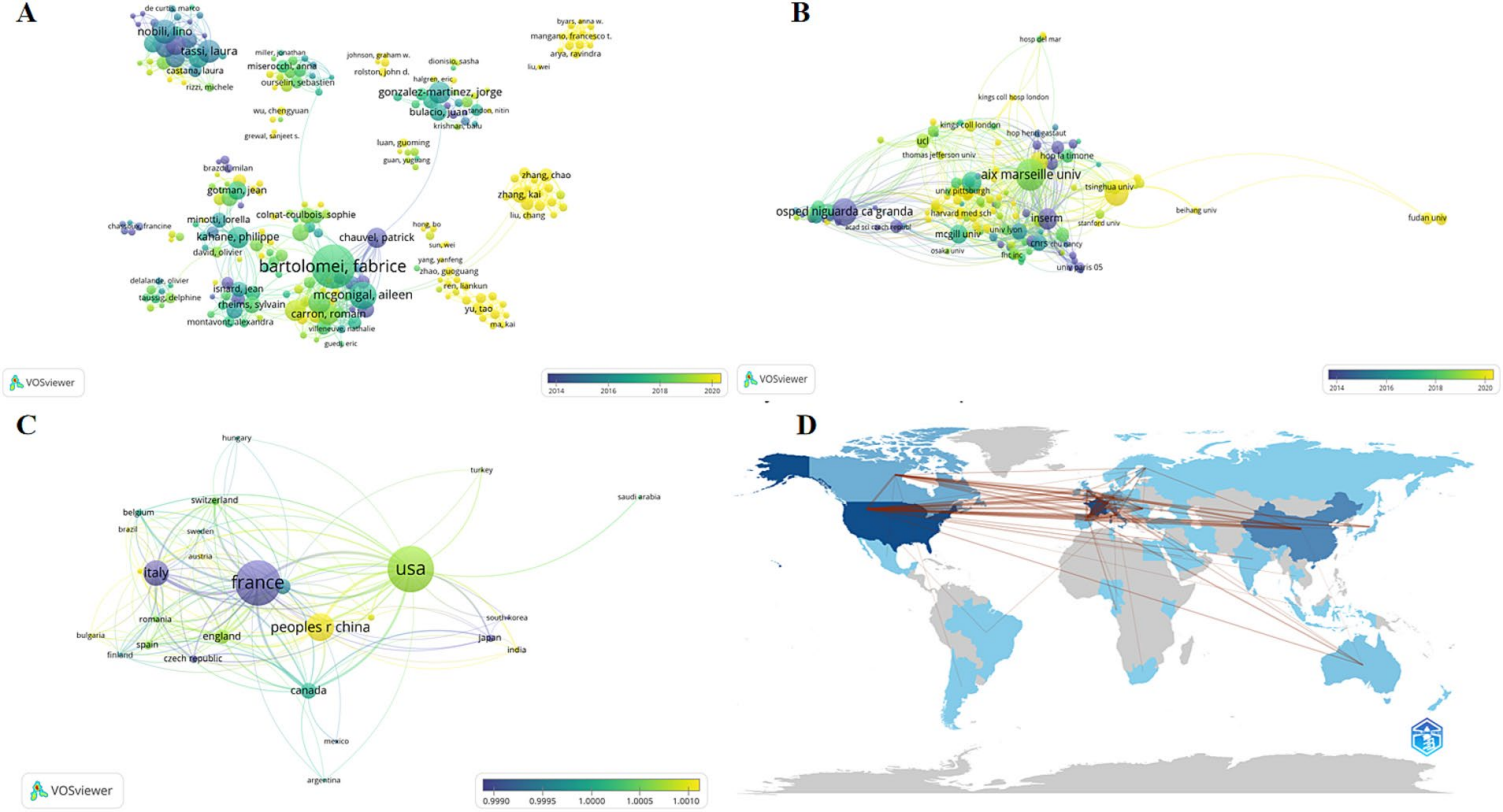
The analysis network of different dimensions of the publications. The figures reflects the authorship **(A)**, institution **(B)**, and country or region **(C)** of all articles included in the analysis. Each node represents an item, with the size of the node indicating the number of published articles; larger nodes correspond to more publications. The color of the node represents the peak publication period, with bluer colors indicating earlier peak periods and yellower colors indicating later peak periods. The lines represent collaborations between items, with the thickness of the lines indicating the strength of the collaborative relationship between two subjects. **(D)** Countries’ collaboration world map by bibliometrix.

The following graph displays each author’s publication volume and citation counts over the years (Figure 3A). In this graph, the size and color of the dots represent relevant information about the articles published by the authors in each year. Larger dots indicate a higher volume of publications, while a darker color signifies a higher number of citations in the counted year. This visualization reveals that Bartolomei F, who has the highest total volume of publications, has consistently maintained high-quality output in the SEEG field in recent years. Not only does he lead in terms of the number of publications (*n* = 123), but his citation counts also suggest his significant contributions to the development of SEEG. Additionally, McGonigal A and Kahane P have also maintained a state of high quality and high output in recent years.

VOSviewer provides a visualized network diagram of authors. In this diagram, larger circles indicate a higher total publication volume for an author, while the color represents the peak period of publication for that author. A bluer color indicates earlier publication dates, and a yellower color signifies more recent publications. According to the results, European scholars led by Bartolomei F and Tassi L were among the first to conduct SEEG research and have guided the mainstream development of SEEG for a considerable time. While in recent years, American scholars like Mangano Francesco T and Chinese scholars including Zhang Kai, Zhao Guoguang, and Yu Tao began to emerge around 2020. Although they do not match the publication volume of the European school, recent hot research topics have seen participation from both American and Chinese scholars. In the diagram, the lines between authors represent collaborative relationships, with thicker lines indicating closer cooperation between the authors connected by these lines. It’s apparent that collaborations between different countries are not very close, suggesting that geographical factors play a significant role in causing a relative isolation in academic collaborations.

### Analysis of institutions

Analyzing various research institutions helps identify leading scientific organizations and hospitals in the SEEG field. This not only facilitates academic exchanges but also serves as a valuable reference for patients seeking related treatments. In our analysis of research institutions using Biblioshiny, the results reveals that nine of the top 10 institutions in the SEEG field are based in France, with UDICE – French Research Universities leading the pack, having published 755 papers. This consortium includes other major contributors like Aix-Marseille Université (428 papers), Université Paris Cité (125 papers), and Université Claud Bernard Lyon 1 (102 papers), all ranked among the top 10 in SEEG research output. Additionally, Assistance Publique – Hôpitaux de Marseille (310 papers), Institut National de la Santé et de la Recherche médicale (INSERM) (255 papers), Centre National de la Recherche Scientifique (CNRS) (171 papers), Ospedale Niguarda Ca’Granda (121 papers), and CHU de Lyon (117 papers) are also among the top 10, highlighting France’s dominance in the field. The only institution outside France in the top 10 is Capital Medical University in China, with 121 publications. This underscores France’s early leadership in academic contributions to SEEG, fostering prominent scholars who have achieved significant recognition (Figure 3B). Meanwhile, recent advancements in SEEG technology in China have brought significant progress, providing East Asian demographic data for research and infusing new vitality into the academic community.

### Analysis of countries of regions

The analysis of publication volumes by country or region can reflect the historical development trajectory of the topic. The data on the number of publications by the institutions’ countries and regions indicates that SEEG research is still significantly influenced by geographical factors and has not yet achieved widespread global application.

Sorted by the number of publications, the top 10 countries are France (288 publications), the United States (275 publications), China (167 publications), Italy (123 publications), Canada (54 publications), Germany (43 publications), the United Kingdom (40 publications), Spain (22 publications), Australia (20 publications), and the Czech Republic (19 publications) (Figures 3C,D). The top 10 countries and regions together account for 1,051 publications, making up 88.5% of all publications. It is clear that the SEEG field exhibits significant regional concentration, with research predominantly centered in developed nations. Developed countries in Europe and America, led by France, hold a definitive global leadership position in SEEG research. Among developing nations, only China ranks in the top 10. This indicates that SEEG has not yet become a diagnostic tool accessible to epilepsy patients worldwide. There remains considerable potential for the expansion and broader dissemination of this technology.

### Analysis of research hotspots

**Figure 4:**
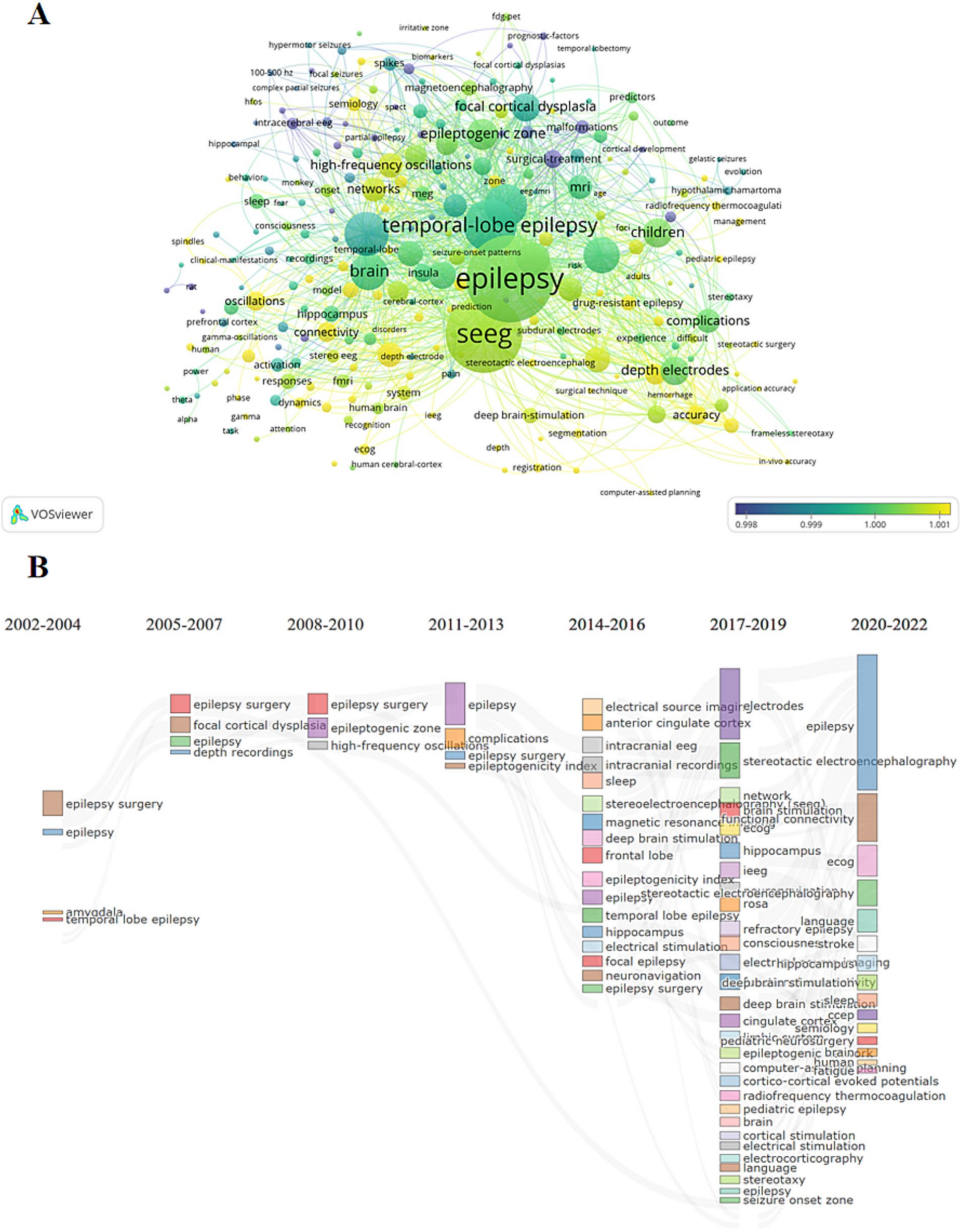
Research hotspot analysis. **(A)** The graph shows the evolution situation of the research hotspots. The elements have the same meanings with those in Figure 2. **(B)** The evolution of research hotspots which divided into three-year intervals. The connections between different keywords represent the evolutionary relationships between them, indicating how research focus areas have changed over time.

By analyzing the keywords associated with articles over time, we gain a visual understanding of how research focuses have evolved in the field. In the graphical representation (Figure 4A), each keyword is depicted as a circle, with colors indicating the peak publication periods for articles associated with those keywords. The more yellow the color, the more recent the period; the bluer the color, the earlier the period. The size of each circle represents the frequency of each keyword across all of the articles.

From its inception, SEEG technology has been closely linked to epilepsy. Initially, SEEG was primarily used to assist in locating epileptogenic foci for surgical interventions and to explore initial methods for collecting intracranial electroencephalographic data. Over time, as the concept of brain networks has been developed and refined, SEEG technology has increasingly been combined with other modalities such as MRI, FDG-PET, and MEG. This integration has been applied to explore brain network mechanisms, establishing a new system for diagnosing and treating epilepsy, reflecting the evolving sophistication and application of SEEG in understanding and addressing complex neurological conditions (Figure 4B).

## Discussion

As a diagnostic tool, SEEG has increasingly played a pivotal role in the field of epilepsy surgery over the past two decades, aided by the development of disciplines such as materials science, computer science, and mechanical industrial manufacturing. Furthermore, it also serves as a crucial means for acquiring intracranial electroencephalograms, providing valuable data for cutting-edge areas such as the mapping of brain networks and the development of brain-computer interfaces. A few of previous retrospective articles have summarized the indications and safety of SEEG since its invention, particularly in its applications within the diagnosis and treatment of epilepsy (21). This article aims to provide a more objective review by leveraging as many publications in the field as possible through bibliometric analysis and database research. From the perspectives of authors, institutions, countries, and keywords, it offers a comprehensive retrospective of the forefront developments in the SEEG field over the past 20 years. Through this review, we hope to project the future development trajectory of this field.

Through the aggregation and analysis of the bibliographic data, it is observable that the total number of published papers involving SEEG in the WOS SCI-EXPANDED database has shown an overall upward trend. From 13 papers in 2002 to 195 papers in 2023, SEEG has gradually garnered increasing attention, and reaching a historical peak in annual publication volume with 195 papers in 2023. It can be stated that SEEG is at the height of its popularity currently.

Through the analysis of journals, it has been found that the magazines publishing related articles all fall within primary categories such as neurosurgery, neurology, and epilepsy. Among them, Epilepsia stands out as the most significant journal in this field, ranking first both in terms of the number of articles published (94 articles in total) and the local impact (H-index = 36) of the articles surveyed. It began publishing relevant articles as early as 1966 and has continued to do so consistently. While among other journals, Brain, although only ranked ninth in terms of the number of publications (28 articles in total), achieves the second position in terms of the impact of its articles (H-index = 25). Additionally, subspecialty journals such as Clinical Neurophysiology, which focuses on neurophysiology, and Neuroimage, which focuses on neuroimaging, also select a significant number of articles in this field for publication. This indirectly highlights the significant contributions of SEEG in the collection of intracranial electrophysiological signals. It also reflects the considerable interest among scholars in the application scenarios where intracranial electrical signals and imaging techniques combines together to interpret the pathophysiological mechanisms of the nervous system, as well as the working mechanisms of the brain under physiological conditions.

The “Authors’ Local Impact” serves as a comprehensive index reflecting the influence of authors within a field, represented by metrics such as the local h-index, local g-index, and the total number of local citations. In the analysis of authors publishing in this domain, Bartolomei F stands out as the author with the highest local impact and also the highest publication volume. Among his publications, the article revealing the relationship between the onset of temporal lobe epilepsy seizures and the thalamus received the most citations (22). This article highlighted the crucial role of increased thalamo-cortical coupling in the loss of consciousness during epileptic seizures and elucidated the importance of the extension of the epileptogenic network to the thalamus in the prognosis of temporal lobe epilepsy seizures. Additionally, his works on different types of epilepsy under various classification systems, such as frontal lobe epilepsy (23), mesial temporal lobe epilepsy (24), and MRI-negative epilepsy (25), through the tracing of seizure origins and mapping of network characteristics using SEEG (26), as well as articles on the prediction of epileptic seizures and the determination of related biomarkers using SEEG (27), also garnered significant citations. This shows that the localization of SOZ and the prediction of seizure onset have always been the focus of scholars around the world. Additionally, Russo GL (h index = 34), Tassi L (h index = 34), Cardinale (h index = 31), and others co-authored the most cited article in this field, which used SEEG to depict the electrophysiological characteristics of FCD and identified three types of FCD (19). In addition to the scholars mentioned above, the achievements of some emerging scholars in this field in recent years also deserve attention. In the topic of preoperative assessment of epilepsy, Mangano and others comprehensively compared the localization effect and the impact on the postoperative seizure prognosis between SEEG and subdural implanted electrodes (28, 29). They proved that epilepsy patients evaluated through these two methods have similar surgical prognoses and similar accuracy in depicting the extent of the language and motor regions. Moreover, the evaluation methods via SEEG also have advantages in terms of surgical damage and the depiction of sensory regions, verifying the superior efficacy of SEEG. While in the topic of SEEG-mediated thermal ablation lesioning for epilepsy foci, Zhao et al. proposed a stereo-crossed radiofrequency thermocoagulation scheme that has been applied to medial temporal lobe epilepsy and insular epilepsy (12, 30, 31). Compared to the traditional ablation method using the same contact, patients treated with this scheme showed significantly better seizure prognosis, and compared to traditional open surgery, there were fewer postoperative complications. Although this scheme has not yet been approved by American Food and Drug Administration, it has already been adopted by several hospitals in China.

The analysis of the institutions and countries of the published papers jointly indicates that Europe, particularly France and Italy, were the earliest to establish the academic system of SEEG. These two countries still remain important producers of academic content in this field today. France has maintained a pivotal position since the beginning of the statistics collected for this article, while Italy held the second position in terms of the number of publications until it was surpassed by the United States in 2019 and then by China in 2021. This also outlines the historical evolution and dissemination path of this technology. Notably, research based on SEEG is mostly concentrated in developed countries, with only a few developing countries like China participating. It seems that the need for expensive surgical equipment and the high cost of electrodes are significant factors limiting further development and application of this technology.

Overall, bibliometrics offers researchers a method to analyze a particular topic, which can review the developmental history, understand hot frontiers, and predict future prospects. The high-frequency keywords that appear alongside the target keywords suggest the historical evolution of this technology, while also outlining the hot topics currently of interest to scholars. Through our analysis, the cutting-edge topics in this field currently mainly include the following aspects.

### The surgical approach and hardware foundation

In the 1960s, SEEG was initially developed solely as a method to complement subdural electrodes in achieving comprehensive and coordinated recordings of the fronto-parietal–temporal cortex and medial temporal structures in patients with temporal lobe epilepsy (32). During this period, the implantation of SEEG primarily utilized a frame-based stereotactic system proposed by Talairach and Bancaud (16). However, it is evident that the implantation of deep electrodes under the guidance of a frame-based system during open surgery presents significant challenges. Subsequently, although stereotactic implantation techniques based on the Leksell framework (33, 34) and frames based on Neuromate (Renishaw, Gloucestershire, United Kingdom) robots have been developed (35), operational complexity and numerous application limitations (e.g., for patients with thin or defective skulls) have restricted the widespread use of depth electrodes. While entering 2010s, with the advancement of computer technology, frameless image guidance systems and robotic guidance systems emerged as alternative methods for electrode implantation (35–37). These technologies gradually replaced the frame-based systems and became the mainstream approach for surgical electrode implantation. Particularly, the advent of surgical robots such as ROSA (Zimmer Biomet, Warsaw, Indiana) (38) significantly broadened the applicability of SEEG and accelerated international research in intracranial electrophysiology. And bibliometric studies have also revealed similar trends: the largest growth in publications on SEEG occurred between 2012 and 2019, coinciding with the period when several of the most widely used surgical robots were consecutively approved by the FDA. Additionally, it is noteworthy that while robot-assisted frameless electrode implantation eliminates the need for physical-level registration, its accuracy in positioning has not significantly diminished compared to frame-based methods. In fact, in some cases, it has achieved smaller implantation errors. The advantages of frameless electrode implantation mentioned above make it a significant alternative to traditional frame-based methods (39, 40).

The evolution of implantation techniques and the advancement of equipment have broadened the range of applications for SEEG. It became clear that the depth electrodes enabled interesting evaluation of not just regional but perhaps network-level involvement of seizure onset and propagation patterns. Correspondingly, implantation strategies have also evolved in response to the changing needs for electrode implantation. The French guidelines on SEEG, published in 2018 in Clinical Neurophysiology by Isnard and colleagues (41), is currently the most widely accepted expert consensus on SEEG. As the representation of traditional electrode implantation strategies, this consensus is guided by the original intentions of SEEG’s inception, sets forth specific principles for electrode implantation: The SEEG implantation should aim to (1) Define the epileptogenic zone, (2) Explore the relationship between the suspected epileptogenic zone and functional areas, (3) Assess the feasibility of surgical resection. With the development of brain network theories, the concept of the thalamus as a critical node in epileptic seizures has become a consensus among many scholars. Consequently, some teams have increasingly focused on the thalamus, implanting electrodes to monitor multiple sites spanning the anterior, middle, and posterior nuclear groups within the thalamus, so that the optimal and personalized thalamic modulation target could be determined to guide the implantation of electrodes for deep brains stimulation (DBS) and reach a better prognosis (42–45).

From the beginning of the 21st century, scholars have shown increasing interest in mesoscopic-level neuronal discharge through Stereo Electroencephalography (SEEG) (6, 46). The advancement of SEEG electrode materials has played a crucial role in this regard. Tracing the history of SEEG development, in its early stages, the electrodes primarily utilized conductive and stable metal materials (6). Subsequently, Felix et al. introduced softer materials like silicone into electrode manufacturing, which significantly increased the electrodes’ flexibility to better conform to brain tissue morphology while reducing damage to surrounding brain tissue (47). The conductive components remained composed of stainless steel electrode wires and platinum contacts. Today, electrodes predominantly made of metal materials still meet most clinical needs, including recording electrical signals and performing ablations (48, 49). However, innovations have also introduced new electrodes using novel alloy materials to achieve higher recording accuracy. Notably, microelectrodes for recording single-cell electrical activity and laminar electrodes for layer-specific recordings in gray matter have adopted platinum-tungsten and platinum-iridium alloys, respectively, as materials for electrode (50–52). However, the foreign body reactions and other minor damages caused by silicone and metal materials can lead to gliosis and neuronal death, extending up to 300 μm from the implantation site, significantly affecting microelectrodes targeting single-cell signal recordings (53). In recent years, researchers have begun exploring more biocompatible electrode materials. Guitchounts and colleagues have used polyethylene-carbon, which is more biocompatible and degradable, for making electrodes that effectively prevent neuronal death caused by long-term implantation, successfully achieving long-term dense neuronal recording *in vivo* (54). Keefer et al. have employed electrochemical techniques to coat traditional electrodes with carbon nanotubes (55). This coating reduces electrode impedance and increases charge transfer, significantly reducing recording noise and enhancing recording accuracy and stimulation intensity in both culture and *in vivo* settings. This has made it possible to use a single electrode to simultaneously record local field potentials, multi-unit activity, and single neuron spiking. Although various novel material electrodes have not yet been applied in humans, and macro electrodes integrated with microelectrodes are solely used to satisfy academic interest, the electrical signals recorded by innovative electrode materials and structures have provided significant insights into normal cognitive functions and the physiology of epilepsy. There is good reason to believe that these technologies will not require much more time before they are truly applied clinically.

### SEEG and epilepsy surgery

Since its inception, SEEG has been closely associated with epilepsy surgery, a relationship underscored by bibliometric analyses where “surgery” appeared as a keyword a total of 174 times in the records. Originally designed to assess epilepsy patients whose seizure foci were difficult to accurately characterize and localize, SEEG technology has evolved to include surgical interventions that utilize the thermal effects between contacts to achieve radiofrequency ablation of suspected epileptogenic foci. This discussion will elaborate on the relationship between SEEG and epilepsy surgery from two perspectives: SEEG-assisted localization of resectable epileptogenic foci and SEEG-based radiofrequency ablation.

As the detection level and accuracy of intracranial electrophysiological signals have improved, spontaneous intracranial EEG has become the gold standard for localizing suspected epileptogenic foci (21, 56). The French guidelines authored by Isnard and others clarify the approach to interpreting ictal EEGs (41): (1) The determination of the seizure onset leads is generally characterized by rapid discharges (low voltage fast rhythms) and is often identified as the seizure onset zone; (2) The definition of the epileptogenic zone often relies on specific pre-seizure changes as the main markers, typically including spikes and slow waves prior to a seizure; and (3) Postictal local electrical suppression or slowing also has diagnostic value for localization. Additionally, the Chinese expert consensus by Zhao and colleagues supplements the determination of seizure origin with low-amplitude fast activity or other forms of seizure initiation and recommends focusing on the dynamic evolution of ictal electrical signals, completing the assessment in conjunction with anatomical structures. This consensus underlines the critical role SEEG plays in guiding precise surgical interventions in epilepsy, enhancing both the accuracy and efficacy of these procedures (57).

In addition to monitoring interictal EEG and waiting for natural seizure occurrences to determine epileptogenic leads after electrode implantation, SEEG can also serve as a tool for identifying epileptogenic areas by administering additional electrical stimulation and observing the electrical feedback from targeted contacts. Beginning in the late 19th century, researchers like Barthlow pioneered intracranial electrical stimulation in the human brain to delineate the functions of different brain regions (58). During World War I, German neurosurgeon Otfrid Foerster first employed intracranial electrical stimulation during epilepsy surgeries to precisely locate epileptogenic foci through electrophysiological feedback differences (59). Subsequently, the use of SEEG for electrophysiological monitoring before surgery to further define the extent of surgical resection became more widespread. This led to the development of two methods for mapping epileptogenic areas: high-frequency repetitive electrical pulse stimulation within a few seconds at 50–60 Hz and single electrical pulse stimulation through intracranial electrodes. According to research published by Valentin and others, preoperative assessment using single-pulse electrical stimulation of related electrodes and observing for post-stimulation anomalies (such as delays and repetitive spikings) to verify whether the implant site is within an epileptogenic focus has shown positive outcomes post-surgery (60). When the resection area exhibits abnormal responses, 96% of patients reach Engel Class I and II outcomes 1 year postoperatively, significantly higher than the 56% without electrophysiological support. However, it is noteworthy that while patients with abnormal activity achieve favorable postoperative outcomes, they only constitute 60% of all patients with refractory epilepsy. Addressing this issue, Matsumoto and colleagues have proposed comparing the magnitude of feedback currents received by contacts outside the SPES stimulation point to assess the suspected SOZ (61). This method is based on two consensus points within the field: (1) The SOZ has a far greater impact on the whole-brain network than other regions, and (2) The SOZ exhibits stronger connectivity and demonstrates a “hyper-coupling” phenomenon within it.

In the 1960s, reports emerged on the use of stereotactic implantation of electrodes for thermocoagulative lesioning of target structures (62). Since the 1990s, in addition to its application in pre-surgical electrophysiological sampling for epilepsy, SEEG has also been used in several small case series to treat intractable epilepsy by inducing focal lesions to disrupt pathological neural network pathways (63, 64). This technique, often known as radiofrequency ablation (RFA) or radiofrequency thermocoagulation (RFTC), involves delivering high-frequency electric currents (above 250 kHz) to generate heat, rapidly raising the temperature of the targeted brain tissue to approximately 45°C, thereby causing local tissue denaturation (65). Initially, thermocoagulation through SEEG involved the use of adjacent contacts on a single electrode (66–68). However, recent advancements in this technique in China have led to significant developments. Xuanwu Hospital of Capital Medical University has introduced a method that utilizes adjacent contacts on the same electrode and nearby contacts on neighboring electrodes (less than 7 mm apart) for stereo-crossed lesioning (30). This approach substantially expands the range of treatable lesions and allows for repeated ablation of the same area using different pairs of contacts, which not only prevents tissue carbonization due to localized high temperatures but also ensures thorough destruction of the target area. Up to now, this protocol has been applied to medial temporal lobe epilepsy (12) and insular epilepsy (31). Both have achieved similar clinical outcomes to the conventional lesionectomy. Additionally, Krasimir et al. attempted to use monopolar for lesioning pathological areas, providing greater flexibility in the choice of SEEG contacts for thermocoagulation. This method offers larger lesion volumes while ensuring flexibility. The discontinuous lesions also avoid the limitations imposed by blood vessels on the lesion range (69).

### SEEG and electrophysiology

With advancements in imaging techniques and the continuous refinement of stereotactic techniques, precise implantation in various intracranial locations has become feasible. This provides clinicians with the opportunity to sample cortical and subcortical structures simultaneously without the need for craniotomy. On one hand, SEEG implantation surgery significantly reduces the risk of postoperative infections compared to craniotomies required for subdural electrode placement, thereby extending the sampling duration and enabling observation of the origins and propagation of natural seizure activity (70). On the other hand, as the stereotactic implantation techniques mature and improve, the precision and stability of electrode implantation in different brain regions have significantly increased. This advancement enables the possibility of multi-electrode, multi-regional sampling across the entire brain, and facilitates the tasks previously challenging such as the sampling from non-adjacent brain regions, bilateral suspected regions, and sulcal cortex. Such improvements are increasingly being applied clinically and provide essential information for identifying the primary epileptogenic zones in multifocal epilepsy (71, 72). And the assessment flexibility provided by these advancements offers clinicians a novel perspective on examining the electrophysiological activity associated with epilepsy. This method of simultaneous electrophysiological assessment across multiple brain regions evolves the theory of focal cortical origins of epileptic seizures into a theory based on brain network dynamics, emphasizing the coordinated origin of seizures across multiple foci.

Over the past decade, as research in disciplines such as neuroscience and neurophysiology has deepened, there has been an increasing demand for *in vivo* collection of electrophysiological signals at the level of single neurons and synapses. This need arises both in the exploration of brain functions and in clinical research into the mechanisms underlying network diseases like epilepsy and Parkinson’s disease. And as materials science and associated manufacturing techniques advanced, it became possible to meet such specific needs. In the 1970s, the application of macro–micro electrodes, which feature micro-wires at their tips for *in vivo* brain electrophysiological recordings, was first reported. These electrodes then enabled researchers to observe high-frequency oscillations in the human hippocampus for the first time (66). Subsequently, the application of large electrodes (with contact surfaces >4 mm^2^ for recording local field potentials) combined with microelectrodes (with contact areas of approximately 0.002 mm^2^ for recording single neuron signals) became more widespread. This composite electrode design allows for the simultaneous recording of clinical electrical signals and single-cell discharges (67, 68). Currently, clinical applications of macro–micro electrodes not only position the micro-wire at the end of a macro electrode but also design some products with micro-wires between the contact points of the macro electrode. This arrangement allows for the collection of electrical signals from different cortical layers at the same time after electrode implantation (50). All of these new electrodes provide researchers with the tools to directly record human brain electrophysiological activities, offering higher sensitivity, selectivity, temporal resolution, and spatial resolution than methods before. They enable the recording of local field potentials at sub-millimeter levels and the sampling of neuronal action potentials, significantly enhancing the ability to record the discharge characteristics of various neural cells and map the functions of different brain regions.

In summary, SEEG, initially developed to address the localization and lateralization issues in epilepsy patients, has evolved to also serve as a medium for focal lesion thermocoagulation and as a vital tool for studying human intracranial physiological states and task-related electrical patterns. According to bibliometric analysis, we speculate that the future of this technology will leverage its flexible implantation sites and the ability to sample deep brain nuclei, extending beyond traditional epileptic focus localization based on anatomical and physiological abnormalities. It is poised to offer insights into the mechanisms, pathways, and treatment modalities for epilepsy and other neurological disorders such as Parkinson’s disease and Alzheimer’s disease from a brain network perspective. Additionally, the development of electrodes and stereotactic surgical robots designed to achieve high temporal and spatial resolution in EEG recording remains a focus of research. Moreover, the availability of more cost-effective electrode materials and surgical robots will play a crucial role in facilitating the broader global adoption of SEEG, particularly in underdeveloped regions. It is worth noting that, although the impact of SEEG on neuroscience research and its significant role in the field of brain-computer interfaces are not highlighted in this article, these aspects are still worth considering by readers as additional factors when comprehensively evaluating this technology.

## Conclusion

This study represents the first global analysis of literature on SEEG using bibliometrics. It aims to elucidate the development trajectory and predict future trends in SEEG research for global researchers. Particularly, the focus is projected to shift toward the mapping of single-unit potentials with microelectrodes under various pathophysiological conditions, and the localization of epileptic foci through integrated brain network theories, subsequently guiding surgical epilepsy treatment plans. And with advances in brain network concepts, SEEG has increasingly integrated with modalities like MRI, FDG-PET, and MEG, contributing to a refined system for diagnosing and treating epilepsy and advancing our understanding of complex neurological conditions. However, there are inherent limitations in the techniques employed in this article. The research database included only the core collections of the Web of Science and was restricted to publications in English, which may have omitted relevant studies not included in the analysis. Also, the conclusions drawn from bibliometric analysis in this article may only apply within the study period, and extending these conclusions beyond this period could potentially be misleading. Additionally, despite using three different bibliometric tools, unavoidable overlaps and similarities in keywords could have influenced the results, indicating a need for methodological improvements in future analyses.

## Data Availability

The original contributions presented in the study are included in the article/supplementary material, further inquiries can be directed to the corresponding authors.
